# Inhibitory Effect of Aconitine on Colorectal Cancer Malignancy via Inducing Apoptosis and Suppression of Cell Motion

**DOI:** 10.5152/tjg.2024.24142

**Published:** 2025-01-01

**Authors:** Xuehui Li, Jianglin Hu, Duan Tong, Taotao Yang, Ming Deng

**Affiliations:** 1 Department of Anorectal Surgery, Caidian District People’s Hospital, Wuhan, Hubei, P.R. China; 2 Department of Anorectal Surgery, Dongxihu District People’s Hospital, Wuhan, Hubei, P.R. China

**Keywords:** Colorectal cancer, *Aconitum*, aconitine, apoptosis, invasiveness

## Abstract

**Background/Aims:**

The incidence of colorectal cancer (CRC) has been increasing in recent years worldwide. Aconitine is a diester diterpenoid alkaloid that exhibits an antitumor role in several cancers. Nevertheless, it remains unclear whether aconitine also has antitumor activity in CRC. This study aims to investigate the effects of aconitine on the malignant behaviors of CRC cells.

**Materials and Methods:**

3-(4, 5-dimethylthiazolyl-2)-2, 5-diphenyltetrazolium bromide (MTT) assay was utilized for cell viability assessment. Flow cytometry, western blotting, wound healing, and Transwell assays were implemented for examining the aconitine effect on CRC cell apoptosis, migration, and invasiveness. Animal experiments were performed to further elucidate aconitine’s effect on CRC tumorigenesis.

**Results:**

Aconitine time- and dose-dependently restrained CRC cell viability but was not cytotoxic to normal colorectal mucosa cells. Aconitine facilitated CRC cell apoptosis and hindered cell migration and invasiveness. Aconitine blocked tumor growth in xenograft mouse models.

**Conclusion:**

Aconitine exerts an anti-CRC effect by promoting cell apoptosis and blocking cell migration and invasiveness.

**Main Points**

- Aconitine was not cytotoxic to normal colorectal mucosa cells.
- Aconitine impaired the viability and promoted the apoptosis of CRC cells.
- Aconitine impeded CRC cell migration and invasiveness.
- Aconitine showed antitumor activity in tumor-bearing mice.

## Introduction

Colorectal cancer (CRC) is the third most prevalent malignancy and ranks second in cancer mortality worldwide.^1,2^ Due to the advances in CRC screening, the incidence and mortality rates of CRC have significantly reduced in people aged 50 and over in recent years.^3^ Inversely, its incidence and mortality rates have been increasing in adults under the age of 50.^4^ As estimated, in 2024, there will be approximately 152 800 new cases of CRC and more than 53 000 deaths from the disease in the United States.^5^ Colorectal cancer occurrence is closely related to genetics, male sex, environmental exposure, and lifestyle factors such as smoking and excessive alcohol intake.^6^ As the clinical symptoms of CRC are nonspecific in the early stages, patients are typically diagnosed at an advanced stage, leading to a 5-year survival rate of only around 14%.^7,8^ Despite the advances in CRC treatments such as endoscopic resection, chemotherapy, and immunotherapy, the clinical outcomes are far from satisfactory,^9^ highlighting the need for novel therapeutic methods for CRC treatment.

Evidence suggests that many molecules and signaling pathways are involved in tumor occurrence and progression. For example, long non-coding RNAs (lncRNAs) can affect CRC cell aggressiveness via the regulation of the JAK/STAT signaling pathway, indicating the potential of lncRNAs as therapeutic targets for CRC.^10^ Guo et al. revealed that ITGB3- and ITGAM-positive exosomes can serve as promising diagnostic and therapeutic target spots for early CRC intervention.^11^ Natural plants and their derived compounds have gained increasing attention in the study of cancer therapy for their prominent inhibitory effects on tumor growth and metastasis.^12^ For example, Baicalein, a flavone, induces ferroptosis in CRC cells by regulating the JAK2/STAT3 signaling transduction.^13^ Moreover, a previous study showed that solamargine has antineoplastic activities in CRC by inhibiting the PI3K/Akt signaling pathway.^14^

Aconitine is a diester diterpenoid alkaloid existing in *Aconitum (Ranunculaceae)*. Aconitine has a relatively narrow therapeutic index due to its violent toxicity, with a lethal dose of 2-5 mg for humans.^15,16^ Nevertheless, many reports have illuminated the pharmacological properties of aconitine, such as analgesic,^17^ anti-inflammation,^18^ and immunoregulation.^19^ Importantly, aconitine is considered a promising therapeutic agent for cancer owing to its antitumor effect. Ji et al. proposed that aconitine enhances pancreatic cell apoptosis and represses tumor cell growth by modulating the NF-κB pathway.^20^ Aconitine promotes reactive oxygen species and enhances tumor cell apoptosis, thereby alleviating the progression of hepatocellular carcinoma.^21^ Additionally, aconitine exhibits antitumor activity in ovarian cancer by elevating estrogen receptor β expression and mediating DNA damage, invasion, and apoptosis.^22^ However, whether aconitine also affects the aggressiveness of CRC is unclear.

Herein, we intended to investigate aconitine functions in CRC *in vitro* and in a xenograft mouse model. We speculated that aconitine might have an antitumor role in CRC by hindering the malignant behaviors of tumor cells. The results may be favorable for developing a new therapeutic agent for CRC.

## Materials and Methods

### Cell Culture

Human CRC cell line LIM1215 and the normal colorectal mucosa epithelial cell line NCM460 (WheLab, Shanghai, China) were incubated in RPMI 1640 medium (WheLab) in a humidified atmosphere (37°C, 5% CO_2_). The medium was supplemented with 1% penicillin-streptomycin (Gibco, Grand Island, NY, USA) and 10% fetal bovine serum (FBS; Gibco).

All animal experiments were performed in line with the NIH Guide for the Care and Use of Laboratory Animals and approved by the Institutional Animal Care and Use Committee of the Hubei Provincial Center for Disease Control and Prevention (approval number: 202305102; date: 23 May 2023).

### Preparation of Aconitine

**Figure 1.**
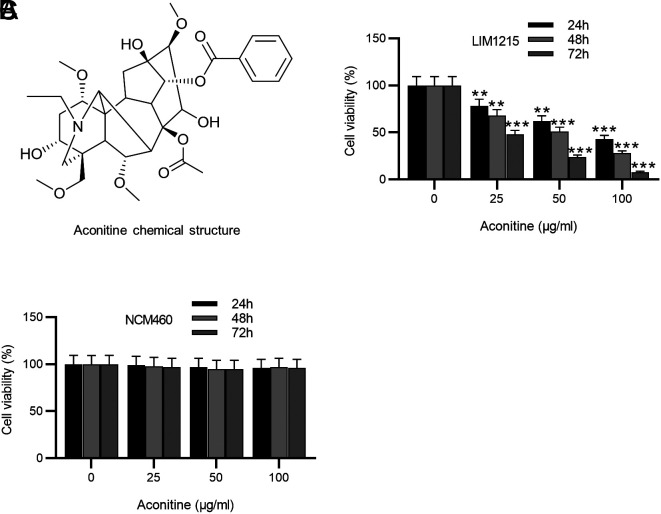
Aconitine restrains the viability of CRC cells but not normal colorectal mucosa cells. A. Chemical structure of aconitine. B-C. MTT assay for evaluating the viability of LIM1215 cells and NCM460 cells treated with different doses (0-100 μg/mL) of aconitine for different periods (24-72 h). **P < .01, ***P < .001.

Aconitine (purity ≥ 95%) was provided by Sigma-Aldrich (A8001, St. Louis, MO). Its chemical structure is displayed in Figure 1A. Aconitine was dissolved in ethanol and diluted to the desired concentrations.

### MTT Assay

3-(4, 5-dimethylthiazolyl-2)-2, 5-diphenyltetrazolium bromide (MTT) assay was utilized for cell viability assessment. LIM1215 cells or NCM460 cells (1 × 10^4^ cells/well) were inoculated into 96-well plates at 37°C for 24 h. After treatment with aconitine (0-100 μg/Ll) for 24, 48, or 72 h, each well was added with MTT solution (10 μL; Beyotime, Shanghai, China) for another 4-h incubation. Next, 100 μL formazan solvent was added to each well. A microplate reader (Thermo Scientific, Waltham, MA) was employed for absorbance evaluation at 570 nm.

### Western Blotting

Radioimmunoprecipitation assay (RIPA) lysis buffer (Solarbio, Beijing, China) was employed for protein isolation from LIM1215 cells. Protein concentration was determined using a BCA assay kit (Solarbio). Protein samples (20 μg) were dissolved by 10% SDS-PAGE, blotted onto polyvinylidene fluoride (PVDF) membranes (Beyotime), and blocked with 5% non-fat milk. The membranes were incubated overnight with primary antibodies (Abcam, Shanghai, China) at 4°C and then with the secondary antibody (1 : 5000, ab97080, Abcam) for 2 h. The blot signaling was detected using BeyoECL Plus (Beyotime), and ImageJ software was utilized for quantifying protein expression. The primary antibodies used are as follows: Bcl-2 (ab32124, 1 : 1000), cleaved-caspase3 (ab32042, 1 : 500), cleaved-poly (ADP-ribose) polymerase (PARP, ab32561, 1 : 1000), Bax (ab32503, 1 : 1000), and GAPDH (ab128915, 1 : 10000).

### Flow Cytometry

An Annexin V-FITC/PI Apoptosis Detection Kit (Beyotime) was employed for cell apoptosis assessment. After exposure to various doses of aconitine (0, 25, 50 μg/mL) for 72 h, LIM1215 cells were digested with 0.25% trypsin without ethylenediaminetetraacetic acid (EDTA), followed by centrifugation at 1000 ***g*** for 5 min and rinsing with phosphate-buffered saline (PBS). Cells were resuspended in 195 μL 1× binding buffer and mixed with Annexin V-FITC (5 μL) and propidium iodide (PI; 10 μL) solution for 10 min away from light. Apoptotic cells were quantified using a flow cytometer (BD Biosciences, Franklin Lakes, NJ).

### Wound Healing Assay

After inoculation into 6-well plates (5 × 10^5^ cells/well) and growing to 90% confluence, a scratch was made on the cell monolayer using a sterile pipette tip. The plates were rinsed with PBS to remove cell debris removal. Cells were exposed to aconitine (0, 25, 50 μg/mL) for 24 h. Cell movements were captured using a phase contrast microscope (Leica Microsystems). Five random fields were chosen to count the cells that migrated across the scratched lines.

### Transwell Assay

LIM1215 cells (1 × 10^5^ cells/well) in serum-free medium (200 μL) containing aconitine (0, 25, 50 μg/mL) were plated in the upper chamber (8 μm pore size, 24-well; Corning Inc., Corning, NY) pre-coated with Matrigel. The lower plate was added with 400 μL of complete medium with 10% FBS. After 24 h, cells in the upper chamber were gently removed with cotton swabs. The invaded cells were fixed in 4% paraformaldehyde (PFA), stained with crystal violet, and examined under a microscope (Leica Microsystems).

### *In vivo* Xenograft Model

Fifteen male BALB/c nude mice (6 weeks, 18-20 g, Cavens, Shanghai, China) were housed under standard conditions. All animal experiments were performed in line with the National Institute of Health (NIH) Guide for the Care and Use of Laboratory Animals and approved by the Institutional Animal Care and Use Committee of the Hubei Provincial Center for Disease Control and Prevention.

After 1 week of acclimatization, the animals were randomly assigned into 3 groups (n = 5/group). All mice were injected subcutaneously with 1 × 10^6^ LIM1215 cells into the murine right axilla. Next, mice in each group were given PBS, 0.1 mg/kg aconitine, or 0.2 mg/kg aconitine orally once daily for 4 weeks. Tumor volume was evaluated every 4 days with the following formula: volume = ½ (length × width^2^). On day 28, all animals were euthanized under anesthesia. Tumor tissues were harvested and weighed, and then fixed in 4% PFA and paraffin-embedded for follow-up hematoxylin-eosin (HE) staining and immunofluorescence (IF) staining of Ki-67.

### Statistical Analysis

Data are expressed as mean ± standard deviation. Each experiment was repeated at least 3 times. One-way analysis of variance (ANOVA) followed by Türkiye’s *post hoc* test was used for comparing differences between groups. Statistical analysis was conducted using SPSS 25.0 software (IBM SPSS Corp.; Armonk, NY, USA), and *P *< .05 indicated statistical significance.

## Results

### Aconitine Restrains the Viability of CRC Cells But not Normal Colorectal Mucosa Cells

To evaluate the cytotoxicity of aconitine, CRC cell line LIM1215 and the non-tumor cell line NCM460 were exposed to increasing doses of aconitine (0-100 μg/mL) for different periods. Notably, aconitine treatment prominently suppressed LIM1215 cell viability, and the suppressive effect was more visible as aconitine concentration increased (Figure 1B). Moreover, at a certain concentration, the inhibitory effect of aconitine on LIM1215 cell viability increased with time (Figure 1B). Nevertheless, no significant change was found in NCM460 cell viability among groups (Figure 1C). The above data indicate that aconitine dose- and time-dependently restrained CRC cell viability but was not cytotoxic to normal colorectal mucosa cells.

### Aconitine Facilitates CRC Cell Apoptosis

**Figure 2.**
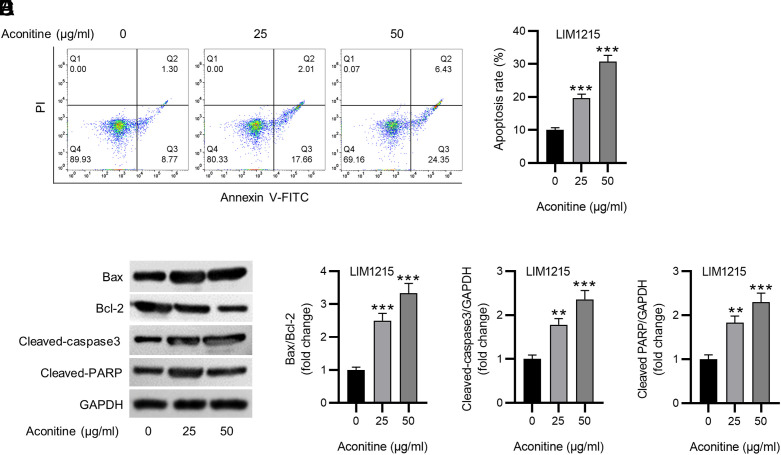
Aconitine facilitates CRC cell apoptosis. A-B. Flow cytometry for detecting the apoptosis of LIM1215 cells under different concentrations (0, 25, 50 μg/mL) of aconitine treatment for 72 h. C-F. Western blotting for measuring expression of apoptosis-related proteins in indicated LIM1215 cells. **P < .01, ***P < .001.

Subsequently, we examined the aconitine effect on CRC cell apoptosis. LIM1215 cells were exposed to 0, 25, and 50 μg/mL aconitine for 72 h. The apoptosis rate of LIM1215 cells was markedly higher in aconitine-treated groups, as depicted by flow cytometry (Figure 2A and B). Likewise, aconitine treatment markedly reduced anti-apoptotic Bcl-2 protein levels, while enhancing levels of Bax, cleaved-PARP, and cleaved-caspase3 levels in LIM1215 cells (Figure 2C). Quantification of the Bax/Bcl-2 ratio, and relative cleaved-caspase3 and cleaved-PARP expression confirmed the above findings (Figure 2D-F). The results demonstrate that aconitine facilitates CRC cell apoptosis.

### Aconitine Hinders CRC Cell Migration and Invasiveness

**Figure 3.**
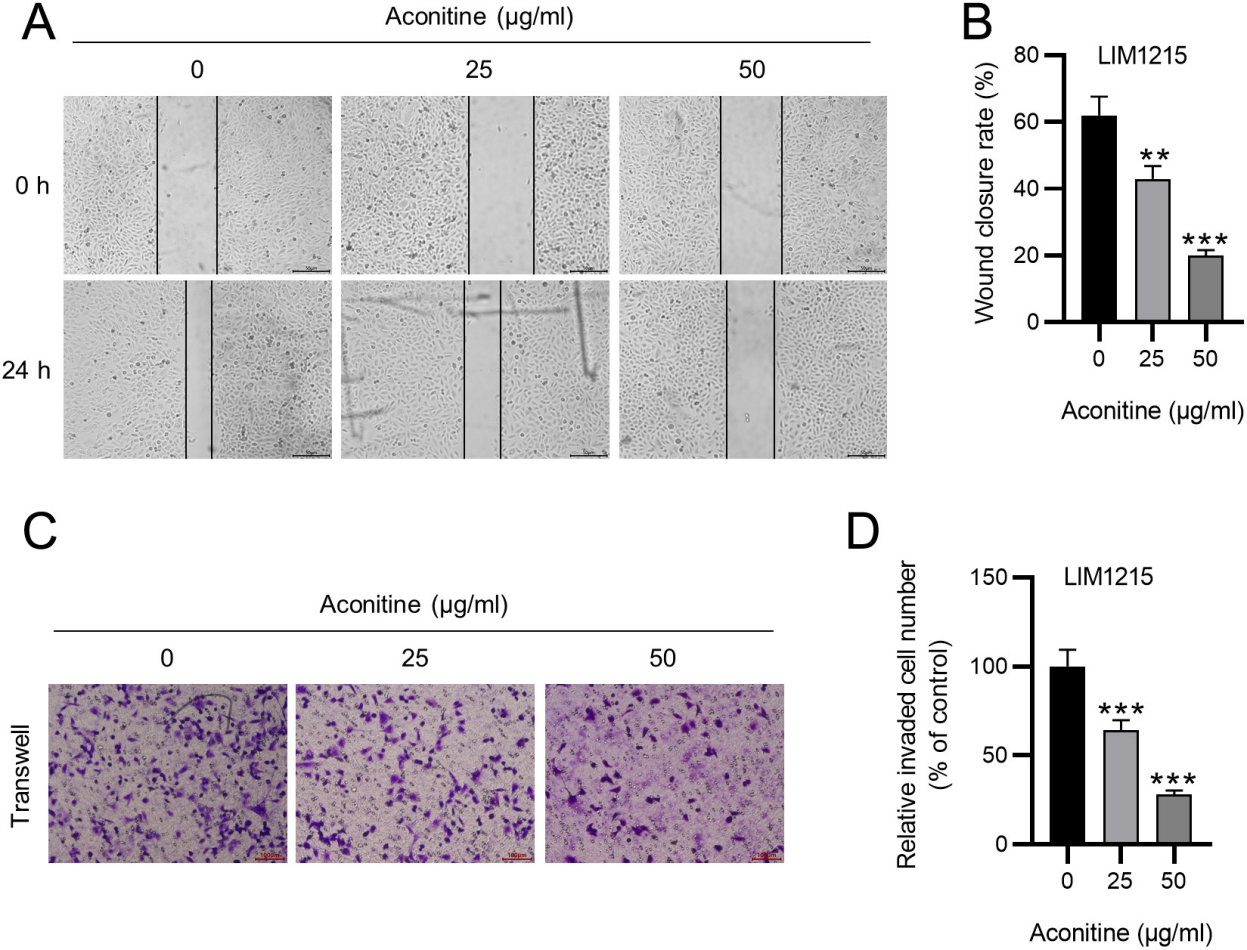
Aconitine hinders CRC cell migration and invasiveness. A-B. Wound healing assay for assessing LIM1215 cell migration in the presence or absence of aconitine. C-D. Transwell assay for determining LIM1215 cell invasiveness under indicated treatment. **P < .01, ***P < .001.

Subsequently, we explored aconitine’s impact on the malignant behaviors of CRC cells. Notably, the number of migrated LIM1215 cells was markedly reduced under aconitine treatment (Figure 3A and B), indicating that aconitine suppressed LIM1215 cell migratory ability. Similarly, aconitine (25 and 50 μg/mL) prominently hindered the invasiveness of LIM1215 cells (Figure 3C and D). The results reveal that aconitine hinders the malignant behaviors of LIM1215 cells.

### Antitumor Effect of Aconitine *in vivo*

**Figure 4.**
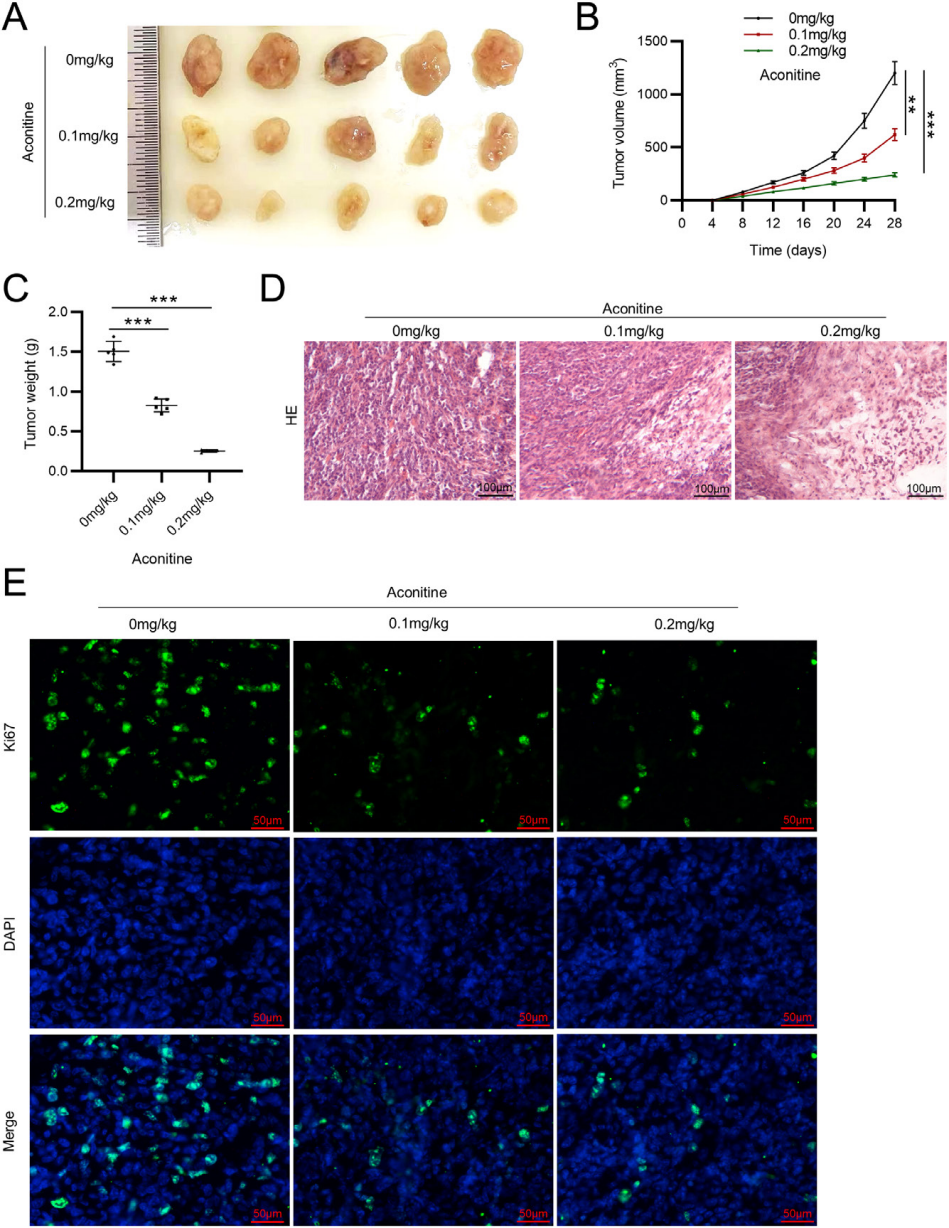
Antitumor effect of aconitine in vivo. A. Tumors isolated from mice in each group. B. The tumor volume of each group was monitored every 4 days. C. Tumors were weighed on day 28. D. Representative images of HE staining for histological analysis of tumors. E. Representative images of IF staining for determining Ki-67 expression in each group. n = 5 mice/group. **P < .01, ***P < .001.

To further elucidate the aconitine effect on tumorigenesis of CRC, BALB/c nude mice were injected with LIM1215 cells, followed by the administration of different doses of aconitine. Tumor volume and tumor weight were measured. As displayed by the results, aconitine administration markedly slowed the growth of tumors in mice (Figure 4A-C). To examine the histopathological changes of tumors, HE staining was conducted. Notably, the tumor cells were densely arranged with large and abnormal nuclei in the group without aconitine treatment, while in aconitine-treated groups, the tumor cells were disorderly arranged and some were necrotic (Figure 4D). Moreover, we examined the expression of Ki-67, a proliferation marker, in tumors of each group using IF staining. As depicted in Figure 4E, aconitine treatment prominently decreased Ki-67 expression in tumors, further confirming that aconitine repressed tumor growth in tumor-bearing mice.

## Discussion

Although the diagnosis and treatment of CRC have improved greatly in recent years, the mortality rate is unacceptably high due to tumor recurrence and metastasis.^23^ Mounting evidence has illustrated that natural plants and their extracts hold great potential in anti-CRC therapy, such as Atractylenolide I,^24^ saponins from Platycodi radix,^25^ and Cardamonin.^26^ Aconitine is a prototypical C_19_-diterpenoid alkaloid from the *Aconitum* plant species which have been widely used in traditional medicinal practices.^27^ Previous reports have revealed that aconitine possesses multiple pharmacological activities and protects against tumorigenesis in several cancers. For example, Wang et al. revealed the suppressive effect of aconitine on ovarian cancer cell growth and migration.^22^ Moreover, previous reports indicated that lappaconitine hydrochloride, a synthetic alkaloid of the Aconite family, triggers apoptosis and suppresses the proliferation of colon cancer cells.^28^ Nevertheless, the specific functions of aconitine in CRC have not been illustrated. Thus, in this study, we examined its impact on the progression of CRC in LIM1215 cells and tumor-bearing mice.

Our results revealed that aconitine time- and dose-dependently suppressed CRC cell viability but had no impact on normal colorectal mucosa cells, indicating that the suppression mediated by aconitine was specific to tumor cells. Apoptosis serves as a crucial process for preventing cancer progression, which is mediated by a balance between Bax (pro-apoptotic protein) and Bcl-2 (anti-apoptotic protein).^29^ Elevation of Bax triggers caspase activation, which induces the cleavage of poly (ADP-ribose) polymerase (PARP), ultimately leading to apoptosis.^30^ This study depicted that aconitine treatment markedly enhanced the apoptosis rate of CRC cells and increased the Bax/Bcl-2 ratio, as well as protein expression of cleaved-caspase3 and cleaved-PARP. These results suggested that aconitine exerted an anti-CRC effect by inducing tumor cell apoptosis, which is consistent with previous reports.^20,21^

Metastatic CRC is characterized by excessive tumor cell migration and invasiveness.^31^ Previous evidence has illuminated that aconitine treatment hinders ovarian cancer cell migration *in vitro*.^22^ In line with this, our results in the study displayed that aconitine prominently restrained LIM1215 cell migration and invasiveness. To further elucidate the antitumor effect of aconitine in CRC, mice were transplanted with LIM1215 tumor cells and administered aconitine. Consistently, animal studies revealed that the administration of aconitine had a significant suppressive effect on tumor growth in xenograft mouse models. Ki-67 is a typical marker for tumor proliferation.^32^ Our results depicted that aconitine repressed Ki-67 expression in CRC tumors, confirming that aconitine might suppress tumorigenesis of CRC. Of note, based on a systematic review and meta-analysis, a previous study showed that aconitine can effectively reduce tumor size and volume, indicating a potent antitumor effect,^33^ which supports our findings in this study.

It is worth noting that our study has some limitations. First, we did not investigate the molecular mechanisms responsible for aconitine-mediated antitumor effects in CRC. Future studies are needed to identify the downstream molecules or signaling pathways involved in the antitumor activity of aconitine in CRC. Moreover, only one CRC cell line (LIM1215) was used for the *in vitro* experiments in this study. To validate the therapeutic potential of aconitine in CRC, further research using other CRC cell lines and more confirmatory experiments is required.

In conclusion, our study is the first to reveal the antitumor role of aconitine in CRC. The results demonstrate that aconitine treatment facilitates CRC cell apoptosis, suppresses cell motion *in vitro*, and inhibits tumorigenesis in the xenograft mouse model. Our findings may provide new ideas for CRC treatment.

## Data Availability

The data that support the findings of this study are available on request from the corresponding author.
